# Influence of Empathy Disposition and Risk Perception on the Psychological Impact of Lockdown During the Coronavirus Disease Pandemic Outbreak

**DOI:** 10.3389/fpubh.2020.567337

**Published:** 2021-01-20

**Authors:** Nicola Grignoli, Serena Petrocchi, Sheila Bernardi, Ilaria Massari, Rafael Traber, Roberto Malacrida, Luca Gabutti

**Affiliations:** ^1^Consultation-Liaison Psychiatry Service, Organizzazione Sociopsichiatrica Cantonale, Mendrisio, Switzerland; ^2^Department of Internal Medicine and Nephrology, Regional Hospital of Bellinzona and Valli, Ente Ospedaliero Cantonale, Bellinzona, Switzerland; ^3^Institute of Communication and Health, Università della Svizzera Italiana, Lugano, Switzerland; ^4^Sasso Corbaro Medical Humanities Foundation, Bellinzona, Switzerland

**Keywords:** COVID-19, isolation, lockdown, psychological distress, risk, empathy, prosocial, ethics

## Abstract

During the current COVID-19 pandemic, and especially in the absence of availability of an effective treatment or a vaccine, the main health measure is neither chemical nor biological, but behavioral. To reduce the exponential growth of infections due to the new coronavirus (SARS-CoV-2) and the resulting overburdening of the healthcare system, many European Countries, parts of the US and Switzerland gradually implemented measures of quarantine and isolation defined as lockdown. This consideration leads to the need to understand how individuals are motivated to protect themselves and others. Recent research suggested that prosocial mental dispositions, such as empathy, might promote adherence to social norms of distancing. Other research conducted during the COVID-19 outbreak indicates, however, that empathy levels might fluctuate according to anxiety linked to the risk of death, and this negatively predicted prosocial willingness. The present protocol proposes a study on whether people's empathic dispositions, interacting with the levels of risk, influence the psychological impact of lockdown. The rationale is that emphatic dispositions, encouraging the acceptance of the lockdown, determine a better psychological adaptation and less distress. One retrospective study will be developed in Switzerland and, if the pandemic conditions force a new wave of lockdown on the population, one prospective study as well. A total of 120 participants will be involved, distinguished by their level of objective risk: (1) high objective risk (COVID-19 positive patients, hospitalized in isolation in post-acute phase); (2) moderate objective risk (COVID-19 positive patients, isolated at home); (3) minimum objective risk (non-positive adults, in lockdown). Measures of perceived risk of being contagious for third parties, empathic dispositions and acceptance of lockdown will be collected. The expected results provide important answers related to the immediate impact of empathic dispositions, effective risk and risk perception on the psychological impact of lockdown during a pandemic outbreak. Data gathered from this study could inform policy makers and public health managers about the best communication strategies that will take into account the various stages of health risk and, in particular, to modulate messages to the population aimed at inducing self-isolation behaviors.

## Introduction

In December 2019, originating in Wuhan, China, a new coronavirus disease (COVID-19) emerged which led to an epidemic of an acute respiratory syndrome (SARS-CoV-2). Within 3 months, the virus had caused more than 118,000 cases and resulted in 4,291 deaths in 114 countries, leading the World Health Organization to declare a global pandemic. The pandemic has led to a massive global effort by local health systems to deal with the cases of infection and to reduce the number of deaths. The most common and useful preventive measures require an increase in hygiene practices (e.g., frequent hand washing, reducing face touching, use of tissues, sanitization of environments). However, in most cases, these measures have not been sufficient, and COVID-19 has forced people to change their habits, from wearing masks in public to physical or social distancing.

On February 24, 2020, Switzerland registered the first case of COVID-19 infection in Canton Ticino, the Italian-speaking part of the country. The number of positive cases increased rapidly in the following days, as did the number of deaths. Three months later, there were 358 confirmed laboratory cases and 19 deaths per 100,000 of the population in Switzerland (1). Neighboring Italy, one of the countries worst affected by the coronavirus, announced 380 confirmed cases and 54 deaths per 100,000 of the population (2). To reduce the exponential growth of infections and the resulting overburdening of the healthcare system, the Swiss Federal Government, like many European Countries and parts of the US, gradually implemented measures to restrict individual freedom (i.e., lockdown for non-health workers).

Other more restrictive measures were implemented in Canton Ticino because of the faster spread of the virus (with an incidence of 958/100,000 confirmed cases on May 25, 2020) due to the proximity of the region with the most affected area of Italy. At first, when the pandemic started to spread across Canton Ticino, contact-tracing measures were applied. Then, when the number of infections increased, those who presented any kind of symptom attributable to the coronavirus infection and people who came into contact with a suspected or positive case of COVID-19 were asked to quarantine at home. Positive COVID-19 patients were isolated either at home or, if so required by their health conditions, in special hospital wards dedicated to COVID-19. For the over-65s, the population group most affected by the virus, the Ticino government strongly recommended not leaving their homes. Care homes were closed to the public. At the end of April, the quarantine and physical distancing measures for non-infected people were progressively reduced in most countries, including Switzerland. Contact tracing and selective isolation in case of contact with a positive case of COVID-19 were re-established for the Swiss population. In the meantime, the scientific community started to fight against COVID-19. Laboratories and researchers in every part of the world have been testing pharmaceutical interventions for COVID-19 [see (3, 4)].

Social behavioral and psychological research has been studying the impact of the pandemic on individuals' well-being and psychosocial functioning. A rapid review of the studies carried out during previous pandemics (e.g., SARS, Ebola, H1N1 influenza) revealed a negative psychological impact on the general population generated by physical isolation and quarantine (5). Short-term effects involved emotional disorders, anxiety, depression, stress, mood decline, irritability, insomnia and PTSD. Whereas long-term effects included increased depressive symptoms, addiction symptoms (i.e., alcohol consumption, substance use) and persistence of avoidance behaviors. The same authors found that the psychological impact of the restrictive measures was boosted by the duration of the quarantine, existing psychiatric disorders, infection fears, financial loss and loss of accessibility to necessities or daily routines, and insufficient information. These pre-COVID-19 results have also been confirmed by further systematic review and meta-analysis of mixed lists of diseases prioritized in public health emergencies (6, 7). This evidence tends to be confirmed by research on confinement conducted during the COVID-19 outbreak (8, 9). Ammar et al. (8) insist on a crisis-oriented interdisciplinary intervention and Serafini et al. (9) suggest focusing on identified protective factors such as resilience and social support or preventive strategies such as effective communication and the provision of adequate psychological services. Public health condition during the epidemic have been acknowledged as a major stressor contributing to an increased risk of psychiatric illness (10). Likewise, regional data on the general population published in Italy highlight the fact that vulnerable people may experience distress during lockdown (11–15).

Evidence emerging from literature has stimulated a debate on what aspects may protect individuals from the negative side-effects of quarantine and isolation. There is a growing body of evidence about communication features that might contribute to mitigating the psychological impact of isolation/quarantine during pandemic outbreaks. To this end, the five review has identified five key public health measures: reducing quarantine duration, providing adequate supplies, providing information, improving communication, protecting vulnerable groups and promoting altruism. These authors also recommend that “public health officials should emphasize the altruistic choice of self-isolating” [5, p. 1].

People's willingness to adhere to preventive public health behaviors is known to be associated with risk perception, which is influenced during the COVID-19 outbreak by various social factors among which are prosocial attitudes (16, 17). Prosocial dispositions have been particularly identified as a core factor in reducing the psychological impact of quarantine during previous pandemic outbreaks (18, 19), and are at the center of an emerging field of research in this domain for adapting public health messages. A recent study in the US (20) confirmed the need for promoting prosocial values: compared to messages that induce fear, prosocial messages capable of arousing a positive emotional state have proved to be more effective in the willingness to accept self-isolation. Another recent study (21) suggests that prosocial mental dispositions, such as empathy, might promote adherence to social norms of distancing, hygiene practices, and ultimately may influence the psychological impact of measures restricting individual freedom. The emphasized rationale is that engaging in physical and social distancing not only protects oneself but also others, especially the most vulnerable, and in this sense is a prosocial behavior. Following this principle, Pfattheicher and colleagues, comparing people from three different countries, demonstrated that empathy increased the motivation for physical distancing.

Other research conducted during the COVID-19 outbreak indicates, however, that empathy levels might fluctuate according to anxiety linked to the risk of death, and this could modulate prosocial willingness (22–25). Perceived risk of infection have been found to be higher in individuals leaving in locations with higher H1N1 incidence and likely to influence the adherence to disease control measures (26). Research on the domain of vaccination for influenza demonstrated that both subjective and objective risk perception were associated with the propensity to take the vaccine (27). Therefore, it seems that, together with empathy, objective and perceived risk may be a variable influencing the adherence to behavioral measures. Perceived risk may be relative to the individual, in other words concern associated with one's own health and survival, which can be experienced by both individuals with or without symptoms. However, the perceived risk can also be relative to third parties: the perception of being a danger to relatives, friends or even simple acquaintances, which again can be experienced by both infected or non-infected individuals.

There are no studies to date that have analyzed whether people's empathic dispositions, interacting with the levels of risk, influence the psychological impact of quarantine/isolation. The rationale that underlines this is that emphatic dispositions, encouraging the acceptance of the quarantine, determine a better psychological adaptation and less distress. On the other side, a high subjective risk perception and a high objective risk should increase the negative psychological impacts of the lockdown because the negative feelings and the emotional concerns caused by the uncertainty of the situation. It might also that the empathic disposition and the objective and subjective risk interact each other in determining the psychological impacts of the lockdown. For clarity of terminology, the term quarantine/isolation has been replaced by lockdown, which is currently used in the international and local media to refer to collective physical and social distancing or isolation during a health emergency.

In particular, the following hypotheses and research questions will be tested:

HP1: high empathic dispositions (vs. low) are associated with the positive psychological impact of the lockdown directly (HP1a) and through the mediation of the acceptance of the lockdown (HP1b).
HP2: high subjective risk perception and high objective risk, independently, are associated with the negative psychological impact of the lockdown.
RQ1: what are the interrelations between risk perception and empathic dispositions on the psychological impact of the lockdown?
RQ2: to what extent does objective risk in combination with empathic dispositions predict the psychological impact of the lockdown?

**Figure 1:**
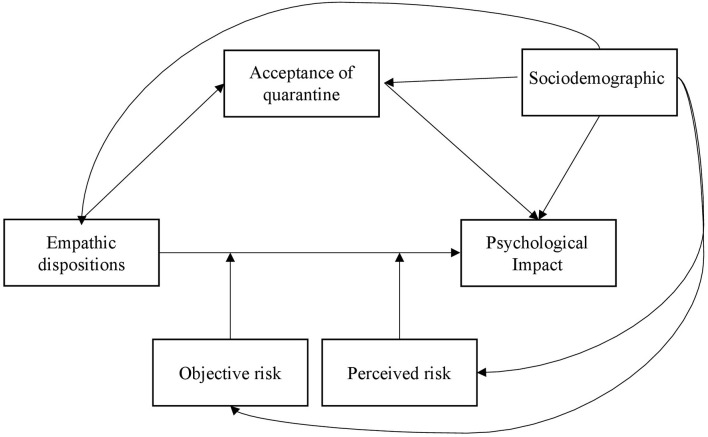
Expected relations between variables.

Figure 1 shows the model tested.

## Methods

This research will take place in Switzerland, specifically in Canton Ticino. The aim will be to measure the effects of the interaction between empathic dispositions and perceived or objective health risk on the psychological impact of lockdown during the COVID-19 outbreak. One retrospective study will be developed and, if the pandemic conditions force a new wave of lockdown on the population, one prospective study will be also developed. The two studies share the same hypotheses and method.

### Participants

A total of 120 participants will be involved in the research. The number of participants has been calculated based on an a-priori statistical estimate (applying GPower v.4), which guarantees adequate statistical power. Retrospective and Prospective studies will involve three groups of participants:

- Group 1: High objective risk (*n* = 60): patients over 18 years of age, tested positive for COVID-19 and hospitalized in isolation in post-acute phase.
- Group 2: Moderate objective risk (*n* = 60): patients over 18 years of age, tested positive for COVID-19 and isolated at home.
- Group 3 (control group): Minimum objective risk (*n* = 60): control group. Persons over 18 years of age, not positive for COVID-19 and in preventive social and physical isolation at home (lockdown).

### Procedure

COVID-19 patients will be recruited through local hospital database on COVID-19 cases provided by Ente Ospedaliero Cantonale (Cantonal Hospital Authority). Participants will complete the survey via a QualtricsTM online link or by paper and pencil. In the retrospective study, participants in groups 1 and 2 will be contacted by phone and will receive the informed consent and questionnaires by post. In the prospective study, group 1 will be contacted directly in the ward and will receive the informed consent and questionnaires to fill out. Group 2 will be contacted by phone and will receive the informed consent and questionnaires by post. Group 3 will be recruited by snowball sampling and data collection will be via QualtricsTM online link.

### Measures

*Demographic*. Self-reported gender, age, living area, marital status, occupation, household composition, will be collected through medical files in EOC's database for Groups 1 and 2 and through specific questions for Group 3.

*Previous health problems*. Self-reported previous diagnosis of non-COVID-19 diseases and/or psychiatric disorder will be collected through medical files in EOC's database for Groups 1 and 2 and through specific questions for Group 3.

*Effective risk exposure (COVID-19 status)*. Participants' COVID-19 status or presence of symptoms attributable to COVID-19 will be collected through medical files in EOC's database for Groups 1 and 2 and through specific questions for Group 3. Questions on COVID-19 status of the household, situation of risk in the household and duration of isolation will be also asked.

*Perceived risk*. One item developed *ad hoc* for this research will evaluate the individual's perception of risk of being contagious for third parties. The item will be formulated according to the measures of perceived relative risk applied in literature [see (28)]. Response options vary from 0 (“no risk”) to 10 (“maximum risk”).

*Empathic dispositions*. Empathic disposition will be measured with three items translated from Pfattheicher et al. (2020). Response options range from 1 (“strongly disagree”) to 5 (“strongly agree”). The items measuring empathy are mixed with three-filler items to reduce demand characteristics.

*Acceptance of lockdown*. Three items will be developed *ad hoc* for this research evaluating participants' acceptance of social and physical isolation measures (lockdown). The item will be formulated according to the measure of physical distancing practice used by Pfattheicher et al. (2020). Labels ranged from 1 (“strongly disagree”) to 5 (“strongly agree”).

*Psychological impact of lockdown (or Distress)*. Psychological impact of lockdown (i.e., Distress) will be investigated with the Italian version of the NCCN Distress Thermometer without the Problem List) (available at: https://www.nccn.org/about/permissions/thermometer.aspx) (29), Patient Health Questionnaire-9 (PHQ-9) (30) and the Generalized Anxiety Disorder 7-item Scale (GAD-7) (31). For both questionnaires, participants indicate how often they have been troubled during lockdown by each symptom, using a four-point Likert scale ranging from 0 (“Not at all”) to 3 (“Nearly every day”).

### Data Analysis

Data will be analyzed through Reliability Analysis, Anova and Ancova, Moderation and Mediation Analysis.

### Ethical Considerations

The study was reviewed and approved by the Cantonal Ethics Committee (N. 2020-01460 /CE3679). Participation is voluntary. All data will be collected and analyzed in an anonymous form. Participants will be debriefed after the experiment. Data will be treated confidentially and used only by the collaborators in the present study for scientific purposes. Participants will receive a written informed consent and will give their consent for their participation.

Evaluating the basic psychological status associated with lockdown during coronavirus could enhance participants awareness of their mental health. The local public psychiatric organization number is included in the Study presentation form. Facilitating access to specific mental health care could be seen as a possible direct benefit for participants in this study.

## Discussion

During a worldwide health risk situation like the one we are facing with COVID-19, especially if effective treatments or vaccines are not yet available for all, the main health measure is neither chemical nor biological, but behavioral. Prosocial behaviors are particularly solicited from the general population when lockdown measures force people to restrict personal freedom and sustain socio-economic and psychological burdens. The results of the present research will provide important answers related to the role of empathic dispositions, objective risk and risk perception on the psychological impact of quarantine during a pandemic outbreak. Data gathered from this study could inform policy makers about the best strategies that will take into account the various stages of health risk and, in particular, to adjust messages to the population.

Behavioral science aims to understand how individuals are motivated to protect themselves and others and how public health managers can promote such self-protecting and prosocial behaviors through specific measures or targeted communication (32–34). Communication at a time of health crisis may induce people to protect themselves and others through fear. However, evidence of the use of fear as a means in communication is inconsistent and often underlines a boomerang effect (35, 36). Individuals might be more prone to respect the quarantine if the communication in time of crisis, such as during the COVID-19 period, stressed the risk of vulnerable people being infected by a virus (37, 38), evoking the individual's empathic tendencies. In fact, some survey research shows that if a restriction of civil liberties (like quarantine and isolation) is oriented to protecting the health of the community and preventing deaths, people tend to accept it (39, 40). Acceptance of the quarantine and isolation measures might decrease the negative impact of the restriction of personal freedom. Measures limiting individual liberty used to reduce the risk of contagion can affect negatively both the mental and physical health of those involved. For the benefit of the wider community, individual freedom is compromised and while isolating sick patients tends not to provoke much concern, collective lockdown or quarantine of healthy people who only might be infected is controversial and tends to provoke ethical concern (41–43). Ethical debate on public health pandemic behavioral prevention and management is open and recent perspectives stresses the value of solidarity and a relational autonomy approach able to ensure a common sense of social justice between the individual self and the others (44).

Finset et al. (45) highlights some elements particularly important in directing communication during a health crisis, such as the one with COVID-19. One of these elements is about the acknowledgment of the psychological impact related to the uncertainty of the situation and fear of infection. In this sense, communicators should express their empathy, demonstrating concern and understanding regarding the impact of the pandemic on individuals' lives. The results of the present research place themselves within this debate with the potential to add several practical considerations. The most important behaviors are well-known – wash your hands regularly, cough in a tissue, keep distance, wear mask, stay isolated if COVID-19 positive – but the way in which the message is implemented is not fully defined. Therefore, results from the present research will make it clear how to adapt the communication of personal and social risk, whether and how to include the empathic concern in the messages in order to maximize preventive behaviors and to decrease the negative psychological impact of quarantine. We also expect that the objective and perceived risk will play a role in determining the relationship between empathic concerns and psychological distress. We expect that the more the situation is uncertain and perceived as a risk for individual health, the more people would be willing to accept message explaining the importance of behavioral measures for their own safety and for the safety of the most vulnerable ones. We can speculate that the experience of those most exposed to risk for their own health could be informative for those less vulnerable. Such shared communication, if adequately promoted through public health messages, could enhance understanding of lockdown measures and ultimately social cohesion. The more health measures and individual restrictions are deliberately adhered to by the population without recourse to communications causing alarm or to coercive measures, the more the negative psychological impact will decrease and the ethical balance between the benefits and risks of personal restrictions will be advanced (46).

The present research has some limitations. First, the retrospective study has disadvantages such as memory bias and difficulty in analyzing the temporal relationship among variables. For this reason, a second prospective study was decided. However, its effective realization is not under our direct control, but depends on the contingent conditions (i.e., second wave). Currently, the retrospective study ensures the possibility to evaluate the state of mind of the individuals under different levels of objective risk without overwhelming people who are already in a difficult situation (i.e., positive to COVID-19). The second main limitation concerns the design. The two studies are cross-sectional and this limits the possibility to derive indications about causality. Another limitation concerns the fact that the measures are self-reported, which may biased the generalization of the results through under-reporting, under-estimating, or having misunderstood the questions.

## Author Contributions

NG, SP, and SB developed the idea and wrote the protocol. IM, RT, RM, and LG contributed to the development of the research questions and checked the final version of the protocol. All authors read and approved the final version of the manuscript.

## Conflict of Interest

The authors declare that the research was conducted in the absence of any commercial or financial relationships that could be construed as a potential conflict of interest.
